# Trajectories of marginal part-time work and risk of depression. Does job or income insecurity mediate the relation?

**DOI:** 10.5271/sjweh.4091

**Published:** 2023-05-01

**Authors:** Helena Breth Nielsen, Jonas Kirchheiner-Rasmussen, Johnny Dyreborg, Ann Dyreborg Larsen, Ida Elisabeth Huitfeldt Madsen, Jacob Pedersen, Anne Helene Garde

**Affiliations:** 1 The National Research Centre for the Working Environment, Copenhagen, Denmark.; 2 Department of Public Health, University of Copenhagen, Copenhagen, Denmark.

**Keywords:** antidepressant, mental health, precarious employment, short working hour, weekly working hour

## Abstract

**Objectives:**

Working few hours a week, known as marginal part-time work, may increase both job and income insecurity, which have been linked to the risk of depression. This study examines the association between marginal part-time work and depression and the mediating role of job and income insecurity.

**Methods:**

We included 30 523 respondents of the Danish Labor Force Survey (DLFS) between 2010 and 2017 and linked them to register-based information on weekly working hours, which was used to identify full-time workers and model group-based trajectories of marginal part-time. These data were linked with survey information on job and income insecurity, and register-based information on hospital-diagnosed depression or redeemed anti-depressant drugs in the following two years. We estimated hazard ratios (HR) by Cox proportional hazards models and conducted mediation analyses to estimate the natural direct and indirect effects using job and income insecurity as mediators.

**Results:**

We identified three distinct trajectories of marginal part-time work: constant marginal part-time work, mobile towards marginal part-time work, and fluctuating in and out of marginal part-time work. Compared with full-time workers, the constant [HR 2.42, 95% confidence interval (CI) 1.83–3.20], mobile (HR 2.84, 95% CI 2.16–3.75), and fluctuating (HR 3.51, 95% CI 2.07–5.97) trajectories all had higher risks of depression. There was no evidence of mediation by either job (HR 1.02, 95% CI 0.92–1.12) or income (HR 0.98, 95% CI 0.89–1.08) insecurity.

**Conclusions:**

We found a higher risk of depression following marginal part-time work. The higher risk was not mediated by job or income insecurity.

Major depression is an important public health concern with an estimated 1-year prevalence of 7% in Europe (1). With around 11 000 new cases of depression each year in Denmark, depression is also among the most common mental health disorders in Denmark (2, 3). The etiology of depression is complex, and likely involves both biological, psychological, and social risk factors (4). The Danish National Board of Health has estimated that depression accounts for 1.7 million more sick days and 751 newly granted early retirement pensions annually among Danish employees (2). Consequently, identifying groups at higher risk of depression may help reduce these numbers by providing the opportunity for focusing interventions aimed at preventing or alleviating depression towards high-risk groups.

Marginal part-time workers, often defined as employees with <15 working hours per week (5, 6), accounted for around 10% of the Danish working population in 2020 (7). The number of marginal part-time workers in Denmark has increased since 2008 and constitutes the largest share of marginal part-time workers in the Nordic countries in 2015 (5, 7). Several factors may influence why an individual takes on marginal part-time work. For instance, marginal part-time work may be part of the transition into the labor market, support a specific lifestyle, or smoothen the transition out of the labor market (8, 9). However, little is known about this group of workers. Results from previous studies suggest that, compared to full-time workers, marginal part-time workers have poorer working conditions (10), less access to training (11), and less variety (12) and complexity (13) of their job tasks. Some marginal part-time workers also face unstable employment, with a lack of power and rights (8). Unfavorable employment quality aspects likely accumulate in marginal part-time work, and some marginal part-time workers are considered to have precarious employments (14). In a newly developed theoretical framework by Bodin et al (14), precarious employment relations have been theorized to affect health through work environment hazards, the experience of precariousness and material deprivation. Recent studies on precarious employment and mental health also showed supporting evidence for a mediating role of work environment hazards (15), and financial strain among men (16). Marginal part-time workers often have variable hours and a lower income, possibly resulting in income insecurity (5, 8). In addition, findings from previous studies suggest that marginal part-time workers also experience higher job insecurity (5, 10, 17) than full-time workers. Reviews have found supporting evidence that low income (18) and high job insecurity (19, 20) are risk factors for depressive symptoms (18–20). Consequently, marginal part-time work could increase the risk of depression through lower income and higher job insecurity. In a previous study, we explored links between marginal part-time work and work environment, job insecurity and health, including depressive symptoms, using a cross-sectional design (10). Results showed that marginal part-time workers more often than full-time workers reported depressive symptoms. However, the one time-point estimate of marginal part-time work combines the effects of all marginal part-time workers at that time-point, yet these could depend on the foregoing path of marginal part-time work in one’s work history.

The aim of this study, is to examine trajectories of marginal part-time work and the prospective association with depression. In addition, we explore if job or income insecurity mediates the association between marginal part-time work and depression. We hypothesize that marginal part-time workers will have a higher risk of depression than full-time workers and that this relation is mediated by job and income insecurity.

## Method

### Study design and data collection

**Figure 1:**
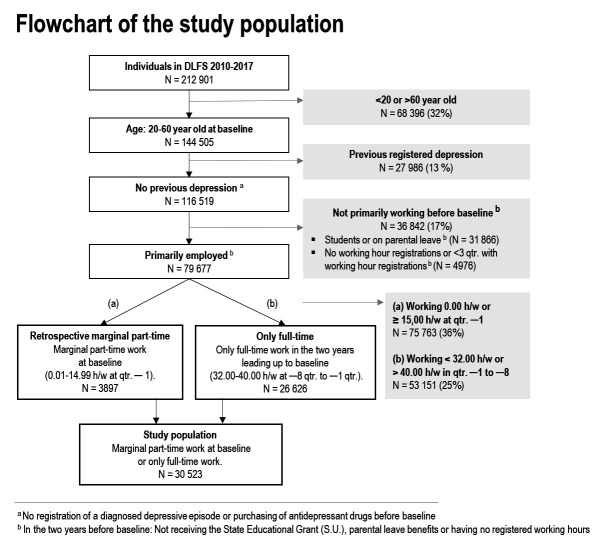
Flowchart of the study population with the retrospective trajectories of marginal part-time work. ^a^ No registration of a diagnosed depressive episode or purchasing of anti-depressant drugs before baseline. ^b^ In the two years before baseline, not receiving the State Educational Grant (SU), parental leave benefits or having regsitered working hours.

**Figure 2:**
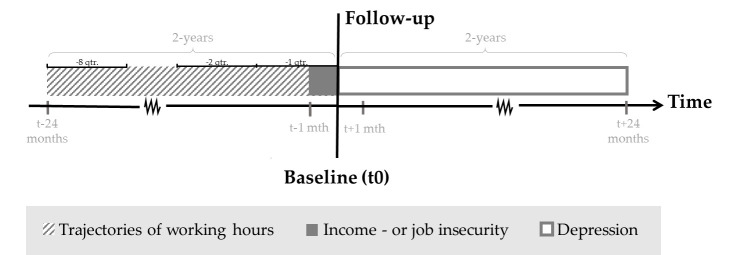
Illustration of the study design. For each participant, the baseline (t0) was set to the DLFS response date. Group-based trajectory modelling was applied on previous working hours evaluated by quarterly average working hours in the two years leading up to baseline (from -8 qtr. to -1 qtr.). Job and income insecurity were assessed at baseline and referred to the past four weeks. Depression was assessed during a two-year follow-up after the baseline.

A total of 30 523 participants from The Danish Labor Force Survey (DLFS) (21) were included and linked with information from Danish national registers on exposure and outcome. Since 1994, DLFS has continually assessed the Danish population’s affiliation with the labor market and contributed to Eurostat’s Labour Force Survey (21). Each year a random sample of around 85 000 Danish citizens, aged 15–74 years, are invited to respond via web interviews or telephone. Each individual is invited to participate in the survey four times over a one-and-a-half-year period. The response rate was 53% in 2013 (23) and around 56% in 2019 (24). In the present study, we included all participants who responded to the DLFS between 2010 and 2017. If an employee had responded to several waves, we used their most recent response. The data of each employee’s DLFS response was used as that individual’s baseline in this study. Figure 1 presents a flowchart with the inclusion criteria for this study: (i) 20–60 years old at baseline, (ii) no prior hospital-diagnosed depression or redeemed antidepressant drugs before baseline, and (iii) primarily employed – excluding students and employees on parental leave as we presume different effects of marginal part-time work on depression than among other workers (10), eg, due to postpartum depression. To handle missing data in the trajectory modelling, employees with <3 registrations of working hours were excluded. Finally, we restricted the population to two groups: (i) employees with marginal part-time work and (ii) employees with full-time work. By restricting the population to these two strata, we were able to capture distinct trajectories of marginal part-time work, as marginal part-time workers only constitute a small share of the population. Employees with marginal part-time work were assessed as working 0.01–14.99 hours/week in the three months leading up to baseline (ie, at -1 quarter (qtr.) in figure 2). In addition, we included all employees with full-time work in the two years leading up to baseline (ie, -8– -1 qtr. in figure 2) as a reference group. Full-time work was assessed as 32.00–40.00 hours/week, which covers the norm of 37 working hours for full-time work in Denmark (25). Employees who solely worked <32.00 or >40.00 hours of work per week in the two years leading up to baseline (ie, -8– -1 qtr. in figure 2) were excluded to ensure a more homogeneous reference group.

### Study variables

*Measures of exposure.* To assess marginal part-time and full-time work, we used a continuous variable of quarterly average weekly working hours for each employee in the two years (eight quarters) leading up to the baseline, see figure 2. Marginal part-time work was assessed by trajectories (see paragraph on statistical analyses), while full-time work was assessed as 32.00–40.00 hours/week in the two years leading up to baseline (ie, -8– -1 qtr. in figure 2). Information on weekly working hours of salaried employees, self-employed and co-working spouses, was derived from the Danish register the Labour Market Account (LMA) without standardization of hours (25), which contains information on labor market status since 2008, including the number of paid working hours (25, 31) of all Danish residents age ≥15 years old (25, 32, 33). Data in LMA derives from several registers, including employer-registered information on all paid jobs of Danish citizens in the e-income register from the Danish Tax Agency (26–28) and LMA is considered to be of high quality (25, 33). In 2013, LMA had <4% missing or invalid information on working hours, which were then imputed (28).

*Measures of outcome.* Depression was assessed by information on hospital-diagnosed depression and redeemed antidepressant drugs. Information on hospital-diagnosed depression was obtained from in- and outpatient somatic or psychiatric hospital visits with the main diagnosis of depressive mood disorder [ICD-10: F32 (depressive episode) or F33 (recurrent depressive disorder)] from The National Patient Register (29) or the Psychiatric Central Research Register (30). The National Patient Register contains information about diagnosis, examination and treatment, together with administrative data, for all somatic and psychiatric hospital contacts in Denmark (31). The quality of the diagnoses in The National Patient Register is of high validity (75.4% for a single depressive episode and 83% validity for a severe episode) (32). As only a fraction of individuals with depression will receive hospital treatment, we further included the use of antidepressants obtained from the purchase of antidepressant drugs (ATC-code category n06a) registered in The Danish National Prescription Registry (33). The Danish National Prescription Registry contains information on purchases of prescription medicine at Danish pharmacies since 1994 (34). All pharmacies are obliged to register the sale of prescription medicine to The Danish National Prescription Registry and the quality of the data is expected to be high (34).

*Measures of mediators.* We used questionnaire data from the DLFS (21) to assess job and income insecurity, which were operationalized in accordance with a previous study on nonstandard work (see supplementary material, www.sjweh.fi/article/4091, appendix 1) (5). The questions were asked at baseline and referred to the four weeks leading up to baseline. *Job insecurity* was assessed by questions covering the risk of losing one’s current job, perceiving one’s current job as temporary, or being unemployed one year ago, as previous unemployment is related to current job insecurity (35). *Income insecurity* is aimed at capturing a perceived inadequate income level. This was measured by questions on applying for, or wanting, additional hours in one’s current job, in a new job or in an additional job.

### Covariates

Covariates were measured at baseline and selected based on previous literature on depression and a directed acyclic graph (see appendix 2). Using information from the DLFS (21), we included the following covariates: sex; age divided into 10-year intervals; education level based on the Danish International Standard Classification of Education (DISCED-15) and categorized as primary, intermediate and higher; industry categorized by the first digit in the NACE codes, the European classification of business activities; and calendar year. Morbidity was assessed by Charlson Comorbidity Index (36) and categorized as any diagnosis within the past five years or none, using The National Patient Register. Cohabitation, categorized as cohabitation or single; and ethnicity, categorized as Danish origin, immigrant or descendant, were all obtained from Statistic Denmark’s Population register (37). Finally, we included the family income, which was divided into quartiles and obtained from Statistic Denmark’s Family income register (38).

### Follow-up

We followed participants from baseline and two years ahead or until one of the following events: death, based on information from the Danish Register of Cause of Death (39); emigration, based on information from the CPR register; endpoint (depression), see figure 2.

### Statistical analyses

The Danish unique personal identification number (40) and the DLFS reference week were used to link data at an individual level.

### Trajectories of marginal part-time work

We used group-based trajectory models (41) to identify trajectories of marginal part-time work. We included employees with marginal part-time work at baseline and assessed their retrospective trajectories of working hours two years back in time (from -1– -8 qtr., see figure 2). Working hours were evaluated as a continuous variable using PROC TRAJ (42) in SAS v.9.4 software (SAS Institute Inc, Cary, NC, USA). We fitted up to ten trajectory groups with a censored normal distribution, starting with all groups set to quadratic equations in accordance with Nagin (2005) (43) (see appendix 3). The number of trajectories was decided by best-fit models, evaluated by the Bayesian Information Criterion (BIC) number, combined with a visual examination of the heterogeneity in the developmental trajectories and the individual’s probability of group membership. The average group posterior probabilities of group membership were all >0.7, as previously suggested (44). Then we tested the best fit by adjusting the polynomial shape for each trajectory, according to the significant level, using higher or lower-ordered parameters.

**Figure 3:**
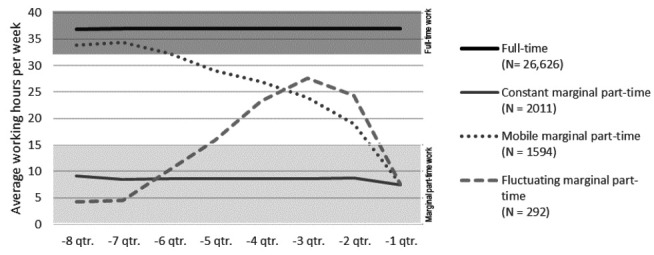
The average number of weekly working hours in the two years leading up to baseline by each retrospective trajectory group.

Consequently, we ended up with ten retrospective marginal part-time work trajectories. These trajectories were combined into three groups based on their pattern in the two years up to baseline (see figure 3): (i) *Constant* – continuous marginal part-time, (ii) *Mobile* – moving towards marginal part-time work, and (iii) *Fluctuating* – fluctuating in and out of marginal part-time work.

### Main analyses

We assessed the associations between each of the retrospective trajectories (constant, mobile and fluctuating) of marginal part-time work and depression using Cox proportional hazards regression models, with time since baseline as the underlying time axis, to estimate hazard ratios (HR) with 95% confidence intervals (CI). An initial visual assessment and test revealed that the Schoenfeld residuals were independent of time and the proportional hazards assumption was fulfilled. All analyses are presented in a crude model with no adjustment; a model 1 including age, sex, education, and calendar year; and a model 2 including model 1 plus industry, morbidity, cohabitation, ethnicity, and family income.

### Mediation analyses

We used the Natural Effect Model by Lange and Hansen (2011) for survival data, based on the counterfactual framework to quantify the mediation between marginal part-time work and depression with job or income insecurity as mediating factors (45, 46). We applied the method by estimating the Natural Effect Cox Model in accordance with the tutorial by Lange et al (47) using 1000 bootstrap repetitions of the procedure to establish the 95% CI. To ensure sufficient power for the analysis, the three marginal part-time trajectories (constant, mobile and fluctuating) were combined into one group.

Data preparation, trajectories and Cox Proportional hazards regression models were performed in SAS 9.4 and mediation analyses were conducted in R using the Medflex packages.

### Supplementary analyses

In addition to assessing the retrospective trajectories of marginal part-time work, we also assessed the prospective trajectories. For the prospective trajectories, we included employees with marginal part-time work (0.01–14.99 hours/week) at two years before baseline (at -8 qtr. in figure 2) and assessed their trajectories of working hours leading up to baseline (from -8– -1 qtr.). This enabled us to capture a possible trajectory from marginal part-time work towards full-time work (prospective mobile) and inspect the robustness of the findings in the overlapping trajectories (constant and fluctuating) of the retrospective trajectories. Using the same methodology as for the retrospective trajectories, we ended up identifying ten prospective marginal part-time work trajectories (see appendix 4). They were then grouped into the three trajectory groups: (i) constant – continuous marginal part-time, (ii) prospective mobile – moving away from marginal part-time work, and (iii) fluctuating – fluctuating in and out of marginal part-time work. In the mediation analysis, the constant, prospective mobile and fluctuating trajectory groups were combined to ensure sufficient power.

### Post-hoc analyses

We assessed the association of marginal part-time and depression by redeemed antidepressant drugs and hospital-diagnosed depression separately. In addition, we assessed the association of marginal part-time and depression stratified by sex and age groups. For these analyses, the three trajectories: constant, mobile and fluctuating marginal part-time work, were combined into one group to ensure sufficient statistical power. For additional details, see appendix 5.

### Sensitivity analyses

We tested the association between trajectories of marginal part-time and depression for (i) survey non-response, by using population-weighted data, and (ii) missing working hour values, by excluding all employees with any missing values of working hours in the two years before the baseline. In addition, we tested (iii) a combined mediating factor of job and income insecurity and finally (iv) mediation using only the constant trajectory in the mediation analysis. All sensitivity analyses were conducted using model 1 adjustments. For additional details, see appendix 6.

## Results

During the two-year follow-up, we identified 502 incident cases (1.6% of the study population), with 487 cases first identified from antidepressant medication redemption and 15 cases first identified from hospital diagnosis.

Descriptive characteristics of baseline covariates by retrospective trajectory group are presented in table 1. We identified 26 626 employees with full-time work and 3897 employees with marginal part-time work at baseline. The constant, mobile and fluctuating marginal part-time work trajectory group comprised 2011, 1594 and 292 employees, respectively. Marginal part-time workers were generally younger than full time-workers (mean 41 versus 46 years of age, P<0.001). Marginal part-time workers were also more often immigrants or descendants (P<0.001). In addition, marginal part-time workers more often had a previously diagnosed illness (P<0.001), and primary education as the highest obtained (P<0.001) than full-time workers.

**Table 1:** Descriptive characteristics of the study population at baseline by the three retrospective trajectories ^a^ of marginal part-time work and full-time work (N = 30 523).

		Marginal part-time work trajectories												Full-time work ^b^	
		Constant ^c^			Mobile ^d^			Fluctuating ^e^			Total				
		N	%		N	%		N	%		N	%		N	%
Sex (women)		1080	53.7		745	46.7		141	48.3		1966	50.4		12 667	47.6
Age (years)															
	20–29	530	26.4		333	20.9		87	29.8		950	24.4		1697	6.4
	30–39	350	17.4		294	18.4		61	20.9		705	18.1		4524	17.0
	40–49	460	22.9		383	24.0		67	23.0		910	23.4		9493	35.7
	50–60	671	33.4		584	36.6		77	26.4		1332	34.2		10 912	41.0
Ethnicity															
	Danish origin	1464	72.8		1400	87.8		213	73.0		3077	79.0		25 126	94.4
	Immigrant or descendant	547	27.2		194	12.2		79	27.1		820	21.0		1500	5.6
Cohabitation															
	Cohabitation	857	42.6		807	50.6		122	41.8		1786	45.8		17 599	66.1
	No cohabitation	1154	57.4		787	49.4		170	58.2		2111	54.2		9027	33.9
Morbidity															
	Previously diagnosed illness ^f^	209	10.4		161	10.1		22	7.5		392	10.1		1333	5.0
	No diagnosed illness	1802	89.6		1433	89.9		270	92.5		3505	89.9		25 293	95.0
Education level															
	Primary	621	30.9		331	20.8		70	24.0		1022	26.2		2712	10.2
	Secondary	770	38.3		843	52.9		141	48.3		1754	45.0		13 182	49.5
	Higher	418	20.8		386	24.2		61	20.9		865	22.2		10 579	39.7
Industry															
	Construction, industry, agriculture, professional service	574	28.5		555	34.8		102	34.9		1231	31.6		7264	27.3
	Public administration, education, health and culture	830	41.3		568	35.6		116	39.7		1514	38.9		10 709	40.2
	Finance, real estate, communication	105	5.2		98	6.2		11	3.8		214	5.5		3198	12.0
	Trade	496	24.7		368	23.1		62	21.2		926	23.8		5441	20.4
Years															
	2010	269	13.4		232	14.6		41	14.0		542	13.9		3126	11.7
	2011	244	12.1		228	14.3		48	16.4		520	13.3		3404	12.8
	2012	269	13.4		218	13.7		39	13.4		526	13.5		3317	12.5
	2013	229	11.4		218	13.7		41	14.0		488	12.5		3471	13.0
	2014	272	13.5		199	12.5		32	11.0		503	12.9		3317	12.5
	2015	271	13.5		207	13.0		44	15.1		522	13.4		3574	13.4
	2016	457	22.7		292	18.3		47	16.1		796	20.4		6382	24.0
Family income															
	1^st^ quartile	1187	59.0		683	42.9		166	56.9		2036	52.2		4854	18.2
	2^nd^ quartile	470	23.4		470	29.5		81	27.7		1021	26.2		6472	24.3
	3^rd^ quartile	216	10.7		264	16.6		28	9.6		508	13.0		7521	28.3
	4^th^ quartile	138	6.9		177	11.1		17	5.8		332	8.5		7779	29.2
Income insecurity (yes)		292	14.5		162	10.1		28	9.6		482	12.4		539	2.0
Job insecurity (yes)		367	18.3		224	14.1		69	23.6		660	16.9		194	0.7

^a^ Paths of working hours across the past two years, which ends in marginal part-time work (0.01-14.99 hours of work per week) at baseline. ^b^ Continuously 32-40 hours of work per week across the past two years before baseline. ^c^ Stable trajectory with constant marginal part-time work. ^d^ Decreasing trajectory going from more hours of work towards marginal part-time work. ^e^ Oscillating trajectory fluctuating in and out of marginal part-time work. ^f^ Within the past five years. Assessed by Charlson’s comorbidity index.

Table 2 shows the HR for the associations between retrospective trajectories of marginal part-time work and depression. All three retrospective trajectory groups with marginal part-time work had higher risks of depression than full-time workers. Compared with full-time workers, the constant marginal part-time work trajectory had a HR of 2.42 (95% CI 1.83–3.20) for depression, the mobile towards marginal part-time work had a HR of 2.84 (95% CI 2.16–3.75), while the fluctuating marginal part-time group had a HR of 3.51 (95% CI 2.07–5.97), after adjusting for age, sex and education, calendar year, industry, morbidity, cohabitation, ethnicity, and family income. In table 3 the natural indirect effects in the relation between marginal part-time work and depression were HR 1.02 (95% CI 0.92–1.12) for job insecurity and HR 0.98 (95% CI 0.89–1.08) for income insecurity. Thus, the mediated proportion for job (1.9%) and income insecurity (-2.3%) were statistically insignificant.

**Table 2:** The associations between retrospective trajectories of marginal part-time work and depression (N = 30 523). [HR=hazard ratio. CI=confidence interval].

Trajectory ^a^		Person years		Cases of depression		Cases per 10 000 person-years		Crude (N= 30 523)			Model 1 ^f^ (N= 30 114)			Model 2 ^g^ (N= 30 089)	
				N		N		HR	95% CI		HR	95% CI		HR	95% CI
Full-time ^b^		52 613		341		64.8		1			1			1	
Marginal part-time															
	Constant ^c^	3797		75		197.5		3.04	2.37‒3.91		2.91	2.23‒3.80		2.42	1.83‒3.20
	Mobile ^d^	3051		70		229.4		3.54	2.73‒4.57		3.31	2.53‒4.33		2.84	2.16‒3.75
	Fluctuating ^e^	552		16		289.9		4.46	2.70‒7.37		4.25	2.52‒7.16		3.51	2.07‒5.97

^a^ Paths of working hours across the past two years before baseline. ^b^ Full-time = 32–40 hours of work per week. ^c^ Constant = Stable trajectory with constant marginal part-time work. ^d^ Mobile = decreasing trajectory going from more hours of work towards marginal part-time work. ^e^ Fluctuating = oscillating trajectory fluctuating in and out of marginal part-time work. ^f^ Adjusted for age, sex, education and calendar year. ^g^ Adjusted for model 1 and industry, morbidity, cohabitation, ethnicity and family income.

**Table 3:** Mediation analyses of job insecurity or income insecurity, in the relation between respective trajectories with marginal part-time work and depression (N=30 523).

Trajectory^a^		Full-time^b^			Marginal part-time^c^										
					Crude				Model 1^d^				Model 2^e^		
		HR	95% CI		%	HR	95% CI		%	HR	95% CI		%	HR	95% CI
Job insecurity															
	Natural direct	1	-			3.24	3.04‒3.44			3.08	2.90‒3.29			2.63	2.45‒2.81
	Natural indirect	1	-			1.03	0.96‒1.12			1.03	0.95‒1.13			1.02	0.92‒1.12
	Total	1	-			3.35	3.26‒3.45			3.17	3.04‒3.32			2.68	2.53‒2.84
	Mediated proportion^f^	-	-		2.66		-3.53‒9.15		2.53		-4.96‒9.91		1.92		-8.44‒11.37
Income insecurity															
	Natural direct	1	-			3.42	3.21‒3.62			3.23	3.05‒3.44			2.74	2.56‒2.93
	Natural indirect	1	-			0.98	0.91‒1.06			0.98	0.90‒1.07			0.98	0.89‒1.08
	Total	1	-			3.35	3.25‒3.45			3.18	3.03‒3.32			2.68	2.53‒2.84
	Mediated proportion^f^	-	-		-1.82		-8.14‒4.94		-1.54		-9.33‒5.48		-2.29		-12.45‒7.43

^a^ Paths of working hours across the past two years before baseline. ^b^ Full-time=32-40 hours of work per week. ^c^ Marginal part-time=All three retrospective trajectories with marginal part-time (0.01‒14.99 weekly working hours at baseline), i.e. includes the Constant, Mobile and Fluctuating marginal part-time trajectory. ^d^ Model 1: age, sex, education, calendar year. ^e^ Model 2: Model 1 + industry, morbidity, cohabitation, ethnicity and family income. ^f^ The mediated proportion is calculated as the coefficients for the natural indirect (log(HR)) divided by coefficients for the total effect (log(HR)) and shown in percentage.

### Supplementary analyses

Results on the associations between prospective trajectory groups and the risk of depression are presented in appendix 4. The association between the prospective mobile trajectory, from marginal part-time work towards full-time work, and depression was HR 1.44 (95% CI 1.03–1.99). The prospective constant (HR 2.16, 95% CI 1.63–2.86) and fluctuating (HR 4.65, 95% CI 2.45‒8.89) trajectories support the results in the retrospective trajectories.

### Post-hoc analyses

In appendix 5, results on the associations between marginal part-time work and redeemed antidepressant drugs showed very similar results as the main analysis (HR range 2.33–3.59). Results on hospital-diagnosed depression also indicated support for the main results based on very few cases. The association between retrospective marginal part-time and depression, stratified by sex and age groups show more than a twofold higher risk of depression among marginal part-time workers compared with full-time workers (HR range 2.20–3.52), in all strata.

### Sensitivity analyses

The results of the sensitivity analyses are presented in appendix 6. The sensitivity analyses supported our main results. Neither non-response nor missing values in the trajectories altered the results markedly. In addition, the results on a combined variable of job or income insecurity as a mediator showed similar results to the main analysis, as did restricting to only the constant trajectory group.

## Discussion

In this large longitudinal study, using register-based information on both exposure and outcome we found that trajectories of marginal part-time work were associated with a higher risk of depression. However, our results did not support that job or income insecurity mediates the relation. The supplementary analyses, post-hoc and sensitivity analyses all supported these findings. This study adds to previous research by evaluating the mediating role of job and income insecurity, assessing trajectories of marginal part-time work, and using register-based information on both the exposure and outcome in a large follow-up design. The results confirmed the hypothesis of a higher risk of depression among marginal part-time workers compared to full-time workers in agreement with the literature on precarious employment, but we did not find any support of this relation being mediated by job or income insecurity.

The current literature on marginal part-time work and mental health is scarce. Our findings align with our previous cross-sectional study on marginal part-time workers, based on a different study population, a point estimate of working hours, and self-reported information on depressive symptoms (10). As such, the two studies are sensitive to different biases; yet both studies point towards a higher risk of depression among marginal part-time workers, strengthening our confidence in this finding. In a broader context, a German cross-sectional study on marginal employment status came to a similar conclusion for women and depressive symptoms but not for men (48). A prospective study found no association between marginal part-time work and depressive symptoms in Germany (49). However, the German studies used different definitions of marginal part-time work than this present study, eg, including German mini-jobs, which is a specific part-time contracts with relatively low income and less social security contributions. Therefore, findings on marginal part-time workers are likely context-specific and relate to the specific definition used.

Several unfavorable employment quality aspects have been linked with marginal part-time workers, including higher job insecurity and poor work environment characteristics compared with full-time workers (5, 10). Precarious employment can be described as an accumulation of unfavorable employment quality aspects (14) and as such, some marginal part-time workers may be considered to be in precarious employments. Our findings are in overall agreement with a recent systematic review on precarious employment, which suggested a negative impact on mental health, in particular for job insecurity (20). A recent register-based Swedish study also showed that employees belonging to low-quality employment trajectories had a higher risk of severe common mental disorder (including depression) as compared with a constant high-quality employment trajectory (50).

Translating the theoretical framework on precarious employment by Bodin et al (14), to marginal part-time work and depression, we expected job insecurity (experience of precariousness) and income insecurity (material deprivation) to mediate the relation between marginal part-time work and depression. Our findings did not find support for a mediating role of job insecurity or income insecurity. This is in line with our previous cross-sectional study, where we observed no indications of mediation by job insecurity. In contrast, two studies from Italy found that economic strain mediated the association between precarious employment and depressive symptoms among men but not women (16). The authors argue that men may perceive job insecurity more strongly than women due to the male breadwinner model (16). The breadwinner model is suggested to be less prominent in Scandinavia due to the large share of women in the labor market and state childcare (51). We also speculate that the apparent lack of mediation through job or income insecurity in our results could be related to the social security system in Denmark, possibly counteracting job and income insecurity among marginal part-time workers with health problems, who are likely to be more prone to depression. This could be evaluated in future studies, possibly by cross-country comparisons.

Marginal part-time work may be voluntary or involuntary. For some, marginal part-time work may be part of a transition into or out of the labor market eg, graduates, immigrants or in relation to unemployment. For others, it may support a specific lifestyle eg, athletes or homemakers, or it may be a way to accommodate a condition eg, due to a chronic disease (5). Consequently, the heterogeneity in the group of marginal part-time workers is likely to be large and our results may cover a mix of risks depending on the actual reason for having marginal part-time work. Therefore, the low number of hours in marginal part-time work is probably not the actual cause of the higher risk of depression. Instead, we argue that marginal part-time work is a good marker when identifying groups of employees vulnerable to depression.

Adding together the distinct shapes of the three marginal part-time trajectory groups and the demographic characteristics (table 1), a potential hypothesis is that the three trajectory groups represent different reasons for having marginal part-time work. The constant trajectory group included a larger share of women, immigrants or descendants, with a low education level, low family income and the highest percentage of income insecurity compared with the other marginal part-time trajectory groups. We speculate that the constant trajectory represents homemakers or employees who struggle to find a full-time job eg, due to negative migration factors such as discrimination in hiring decisions (52). The constant trajectory group is also more often diagnosed with an illness, which could suggest that the employees are not able to take a full-time job due to a chronic condition. The retrospective and prospective mobile trajectory groups were more often of Danish origin and had a higher education level and family income compared with the other marginal part-time trajectory groups. We speculate that the mobile trajectory groups represent employees that due to illness or a traumatic event had to reduce their working hours (retrospective) and when feeling better return back to full-time work (prospective). The retrospective mobile trajectory group were also a bit older, possibly representing those transitioning to retirement, while the prospective mobile trajectory group were a bit younger and could be transitioning into the labor market through internships. Finally, the fluctuating trajectory group were younger, often immigrants or descendants and had a low family income, compared with the other marginal part-time trajectory groups. We speculate that this group represent employees who have difficulties getting into the labor market and holding on to a job eg, due to negative migration factors. These hypotheses on reasons for having marginal part-time work needs to be assessed in future studies. The mediation statistical analyses were based on a combination of the three marginal part-time trajectory groups due to power. However, the mediating effects of job and income insecurity could differ by trajectory group and reasons for having marginal part-time work. Thus, future studies also need to look more into different potential mediating factors related to different trajectory groups. Finally future studies should more thoroughly evaluate the risk of depression and marginal part-time work in combination with other statuses eg, partly retirement, partial sick leave, training, voluntary work, informal care or being a business owner.

### Strengths and limitations

Our findings should be considered in light of this study’s strengths and limitations. One major strength is the large sample size, which covered a random sample of the Danish working population. Another strength is the use of register-based information on both previous working hours and depression. Thus, information is collected independently of this study’s aim and recall and attrition bias is constrained, as follow-up information was register-based (53). The sensitivity analyses with population weights supported our main findings, as such the moderate response rate in the DLFS did not appear to have biased the results markedly.

The use of trajectories is a powerful data-driven way of identifying distinct trajectories of marginal part-time work. By using trajectories of marginal part-time work across two years, we have refined the exposure measure and expect to have reduced the risk of misclassification of exposure compared with using only a single-time point estimate. Using best fit to assign the marginal part-time trajectories means, we will have more contrast in the exposure groups, than in the reference group of full-time workers, yet misclassification between marginal part-time work and full-time work is expected to be diminishing small. We had no information from general practitioners or therapists outside hospitals, which may lead to an underestimation of cases. Yet, the majority of the cases were identified from the redemption of antidepressants, which indicates that most cases are due to consultations with general practitioners. Anti-depressants are used to treat other mental disorders than depression, which will lower the sensitivity for depression. A recent study on indications of prescribed antidepressants drugs in Denmark among ≥65 years old, showed depression as the most frequent indication with 58%, followed by missing or unspecified indication (28%), tranquillizers (7%) and anxiety (6%) (54). There is a risk of bias due to selection mechanisms from differences in treatment-seeking behavior, doctors’ prescription behaviors, redemption as well as the type of treatment that people seek. Some companies offer employees private health care insurance including private therapy. Supposing that full-time workers have better access to private therapy through company benefits and higher income, they are likely more prone to seek out private therapy for mild depressions, while marginal part-time workers may need to go to the doctor and get a prescription instead. Therefore, we cannot rule out that this could result in fewer registered cases of mild depression among full-time workers in this study. However, in moderate-to-severe depression, it is recommended to use anti-depressants in the treatment (55). Consequently, we find it reasonable to assume that the large majority of the moderate-to-severe cases of depression are included for both marginal part-time workers and full-time workers.

We excluded individuals with a prior diagnosis of depression, limiting the risk of reverse causation. However, some employees may change their working hours due to early signs of depression before an actual diagnosis. The mobile trajectory, where employees moved towards marginal part-time work, likely illustrates this. Yet, the results from the constant trajectories, with marginal part-time work for two years before baseline, also showed a higher risk of depression as did the prospective mobile trajectory moving from marginal part-time to more hours of work per week.

Analyses were adjusted for several confounders, yet residual confounding may still be at play including time-varying confounding and morbidity where we only had a crude measure based on diagnoses from hospital visits. Also, having small children at home or experiencing a traumatic event, such as a divorce or loss of a close relative, could be potential confounders. Yet we expect that the exclusion of those on parental leave and adjusting for age and cohabitation would reduce potential confounding from having small children. Unknown and unmeasured confounding could also have violated the assumptions of the mediation analyses, which is a general limitation in most observational studies.

The estimates on the individual marginal part-time trajectories should be interpreted with caution due to the low number of employees in some of the trajectories. This also encumbered a solid comparison of the estimates across the different marginal part-time trajectories. However, the results showed a consistent and strong association across different trajectories of marginal part-time work, both when assessing previous working hours prospectively and retrospectively. We expect the social and societal, political and economic context to affect these results (56) and as such, the findings may only be generalized with caution to other Scandinavian countries.

In conclusion, this study finds that workers with marginal part-time work in the previous two years had a higher risk of depression than full-time workers. We found no support for job or income insecurity mediating the relation. Workers with marginal part-time work in the past two years can be considered a high-risk group in terms of depression. Further studies are needed to confirm these results and distinguish the different groups with marginal part-time work and their risk of depression.

## Supplementary material

Supplementary material

## Financial material support

The Velliv Association (Velliv Foreningen) funded this study and took no part in the design, the analyses, and the preparation of the manuscript or the decision to publish this manuscript.
