# Supplementary material for: Trajectories of marginal part-time work and risk of depression. Does job or income insecurity mediate the relation?
Source: Scand J Work Environ Health. 2023 May 1;49(4):271–82. doi: 10.5271/sjweh.4091 (PMC10713993; doi:10.5271/sjweh.4091)
Supplement: Supplementary material [file SJWEH-49-271-S001.pdf]

Trajectories of marginal part-time work and risk of depression. Does job or income insecurity mediate the relation?[1]

by Helena Breth Nielsen, PhD,[2] Jonas Kirchheiner-Rasmussen, MSci, Johnny Dyreborg, PhD, Ann Dyreborg Larsen, PhD, Ida Elisabeth Huitfeldt Madsen, PhD, Jacob Pedersen, PhD, Anne Helene Garde, PhD

1. *Supplementary material*
2. *Correspondence to: Helena Breth Nielsen. National Research Centre for the Working Environment, Lersø Parkallé 105, 2100 Copenhagen Ø, Denmark. [E-mail: hbn@nfa.dk].*

Appendix 1: Mediating factors

Questions from the DLFS on job and income insecurity and the categorization.

1.1.1 Job insecurity - risk loosing job, temporary job or previous unemployment

Job insecurity was assessed as a combination of questions on: 1) Risk of losing job or having a temporary job and 2) Unemployment one year before the survey. Job insecurity was categorized as: *Yes* (yes to at least one of the two); *No* (no to both – or no and missing); *Missing* (missing in both).

Risk of losing job or having a temporary job
Based on the question "What is the main reason for looking for another job? Choose only the most important reason" (Statistic Denmark variable: LOOKREAS)

Yes: (1) risk losing or ceasing current employment (or knowing that this will happen); 2) considers the current work to be temporary)

No (all others)

Missing (blank)

Unemployment one year before the survey

Based on the question "What do you mainly consider yourself a year ago?" and age (Statistic Denmark variable WSTAT1YR)

Yes: (2) unemployed

No: (1) Performed a job or occupation, including unpaid work in a family, employment as an apprentice or paid trainee, etc; 3) Pupil, student, in continuing or further education, unpaid trainee ;4) Retired or prematurely retired or ceased to run a business; 5) Permanently incapacitated; 6) In compulsory military service; 7) Domestic; 8) On cash benefits (not as available) and other persons who were out of work

Missing: (blank or 9) under 15 years of age or older than 74 years old)

1.1.2 Income insecurity - wishing or seeking more work hours

Income insecurity was assessed as a combination of questions on: 1) Wishing for more hours and 2) Applying for more hours. Income insecurity was categorized as: *Yes* (yes to at least one of the two); *No* (no to both – or no and missing); *Missing* (missing in both).

Wishing for more hours

Based on the questions: “Even though you have not applied for work within the specified 4-week period - ie. from the XX to the XX - would you like to work more hours a week? It could be in your current job, by changing jobs or by getting an extra job”, “How many hours a week do you wish to work in total? This includes your main job and any side job. ”, "How many hours a week do you normally work in your side job? The hours are stated without any meal breaks.", "How many hours a week do you normally work in this job?", “During these 4 weeks - from the XX * to the XX * - have you applied to get more hours in your current job?” (Statistic Denmark variable: WISHMORE)

Yes: (1) yes (Number of desired working hours is higher than current number of working hours (in primary plus secondary jobs); The main reason for looking for another job is to find a job with more working hours; Yes, wish to have more hours, in a second job; Yes, wish to have more hours, in another job; Yes, wish to have more hours, but only in current main job or side job; Yes, wish to have more hours, it does not matter how; Yes, applied for more hours in current job)

No: (all others))

Missing: (not relevant or blank)

Applying for more hours
Based on the question “What is the main reason for looking for another job? Choose only the most important reason” (Statistic Denmark variable: LOOKREAS)

Yes: (3) seeking an additional job to supplement current number of work hours; 4) seeking a job with more hours than the current job)

No (all others)

Missing: (blank or not relevant)

Appendix 2: Covariates

A2.1: Simplified Directed Acyclic Graph of marginal part-time work and depression

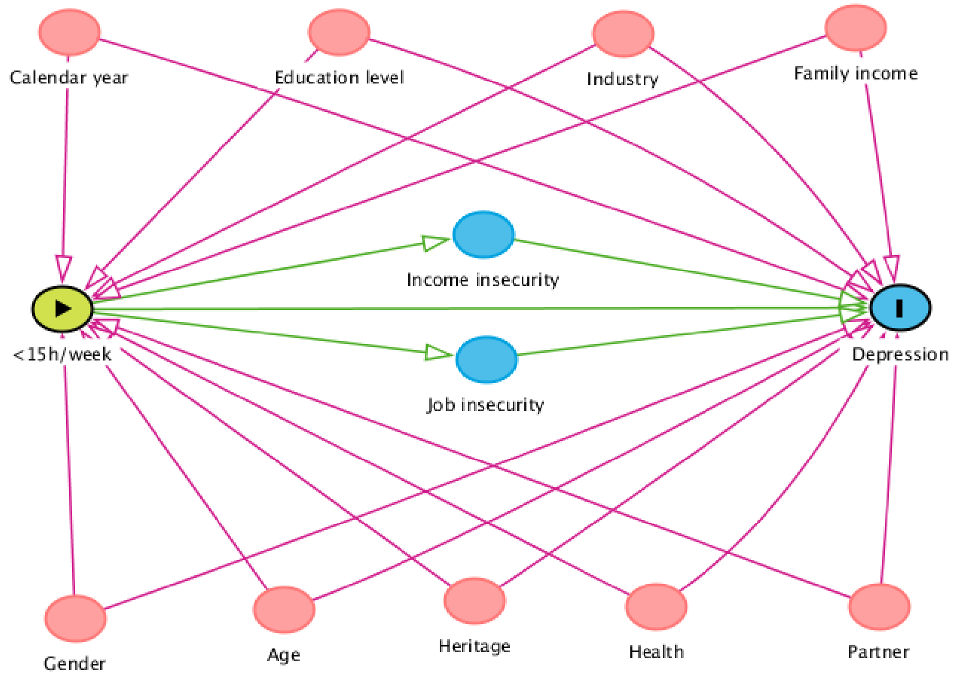

Figure A2.1: *Simplified DAG of marginal part-time work on depression created with Dagitty* (1). *Minimal sufficient set of covariates to estimate the total effect of marginal part-time work on depression: age, sex, calendar year, education level, industry, cohabitation, ethnicity, health, family income.*

exposure, outcome, ancestor of exposure, ancestor of outcome, ancestor of exposure *and* outcome, adjusted variable, unobserved (latent), other variable, causal path or biasing path

Literature relating depression to: Sex (2), Age (2), Education level (3), Industry (4), Calendar year (5), Morbidity (6), Cohabitation (7).

A2.2: Categorization of covariates

Covariate	Variable (register)	Categorization	Codes	Description
Education level	Highest attained education:	Primary	1	Primary education
	Hfaudd translated with DST format		2	Lower secondary education
	audd_level_l4_l1 to ISCED codes	Intermediate	3	Upper secondary education
			5	Short cycle tertiary
	(udda)	Higher	6	Bachelor or equivalent level
			7	Master or equivalent level
			8	Doctoral or equivalent level
		Missing	9	Not elsewhere classified
Industry	Industry with highest number of working hours in: arb_hoved_bra_db07 translated with DST format DB07_L1L5_KT to main groups based on the first digit in the NACE codes and grouped.	Construction, manufacturing and agriculture	A	Agricultura, foresty and fishing
			B	Mining and quarrying
			C	Manufacturing
			D	Electricity, gas, steam and air conditioning supply
			E	Water supply; sewerage, waste management and remediation activities.
	(The Labour Market Account)		F	Construction
			M	Professional scientific and technical activities
			N	Administrative and support service activities
		Transportation and trading.	G	Wholesale and retail; Repair of motor vehicles and motorcycles
			H	Transportation and storage
			I	Accomdation and food service activities.
		Finance and communication	J	Information and communication
			K	Financial and insurance activities
			L	Real estate activities
		Public administration, education, health and culture	O	Public Administration and defence compulsory and social security
			P	Education
			Q	Human health and social work activities .
			R	Arts, entertainment and recreation
			S	Other service activities
			T	Activities of households as employers; undifferentiated goods- and services-producing activities of households for own use.
			U	Activities of extraterritorial organization and bodies.
		Missing		999999
Sex	Sex	Men	1	Men
		Women	2	Women
	(The Danish Labor Force Survey)			
Age	Age in categories	20 -29	1	Between 20 – 30 years old.
		30-39	2	Between30 – 40 years old.
	(The Danish Labor Force Survey)	40 - 49	3	Between 40 – 50 years old.
		5060	4	Between 50– 60 years old.
Morbidity	Charlson Comorbidity Index: Hospital registration of one of the following ICD-10 diagnoses in C_diag during the past 5 years.	None	0	None
		Any	1	One of the following ICD-10 diagnoses in the past 5 years: I21- I23; I50; I110; I30; I32; I70 - I74; I77; I60 – I69; G45; G46; G30; J40-J47; J60-J67; J68.4; J701; J703; J841; J920; J961; J982-

	(The National Patient Register)			J983; M05; M06; M08; M09; M30–M36; D86; K221; K25–K28; B18; K700-K703; K709; K71; K73; K74; K760; E100;E111; E119; G81; G82; I12; I13; N00-N05; N07; N11; N14; N17–N19, Q61; E102–E108; E112-E118; C00–C75; C91–C95; C81–C85; C88; C90; C96; B150; B160; B162; B190; K704; K72; K766; I85; C76–C80; B21–B24.
Cohabitation	Having a cohabitation: civst	No cohabitation	E	Widow/widower
			F	Divorced
			L	Longest-living of two cohabitations
	(Population)		O	Broken cohabitationship
			U	Unmarried
		Cohabitation	G	Married (incl. separated)
			P	Civil cohabitationship
Family income	Family income in quartiles: FAMDISPONIBEL_13 by the distribution of Family income in study population.	1. quartile	1	0–25 per centile in family income
		2. quartile	2	25–50 per centile in family income
		3. quartile	3	50–75per centile in family income
	(Family income)	4. quartile	4	75–100 per centile in family income

Abbreviations: ISCED = The International Standard Classification of Education, NACE = The Statistical Classification of Economic Activities in the European Community

Appendix 2.3: Charlson Comorbidity Index

Included diagnosis in Charlson Comorbidity Index (8-10)

Condition	ICD-10 codes
Myocardial infarct	I21–I23
Congestive heart failure	I50; I110; I30; I32
Peripheral vascular disease	I70–I74; I77
Cerebrovascular disease	I60–I69; G45; G46
Dementia	G30
Chronic pulmonary disease	J40–J47; J60–J67; J68.4; J701; J703; J841; J920; J961; J982–J983
Connective tissue disease	M05; M06; M08; M09; M30–M36; D86
Ulcer disease	K221; K25–K28
Mild liver disease	B18; K700-K703; K709; K71; K73; K74; K760
Diabetes	E100;E111; E119
Hemiplegia	G81; G82
Moderate or severe renal disease	I12; I13; N00–N05; N07; N11; N14; N17–N19, Q61
Diabetes with end organ damage	E102-E108; E112–E118
Any tumor	C00–C75
Leukemia	C91–C95
Lymphoma	C81–C85; C88; C90; C96
Moderate or severe liver disease	B150; B160; B162; B190; K704; K72; K766; I85
Metastatic solid tumor	C76–C80
AIDS	B21–B24

Appendix 3: Retrospective marginal part-time trajectories

The retrospective trajectories were identified among all marginal part-time workers at baseline (at -1qtr. in figure 2), N = 3897 employees. Group based trajectories of marginal part-time were calculated based on quarterly average weekly working hours across the two years leading up to baseline (in figure 2: from -8 qtr. to -1 qtr.).

A3.1 Different trajectory grouping of the retrospective marginal part-time trajectories

---

One group

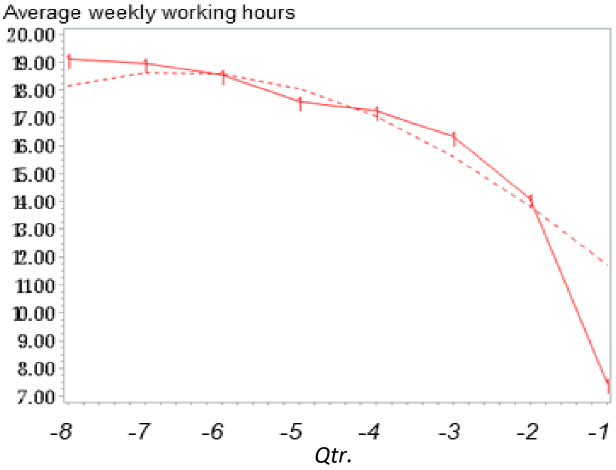

Two groups

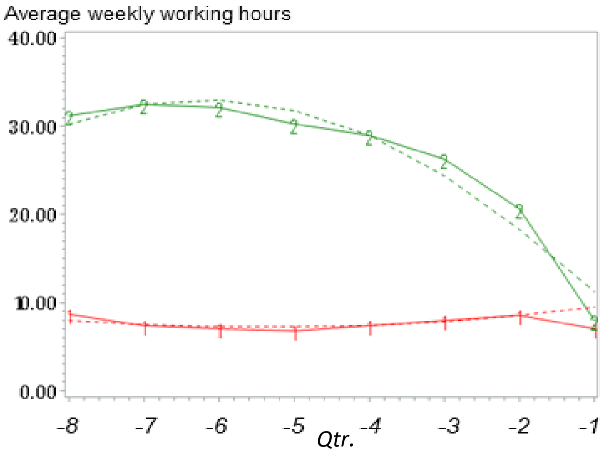

Three groups

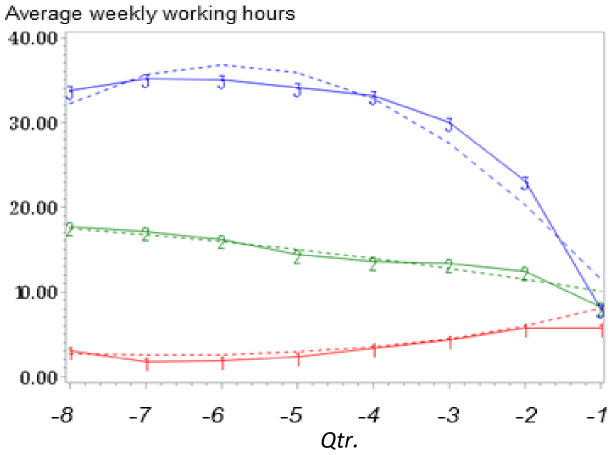

Four groups

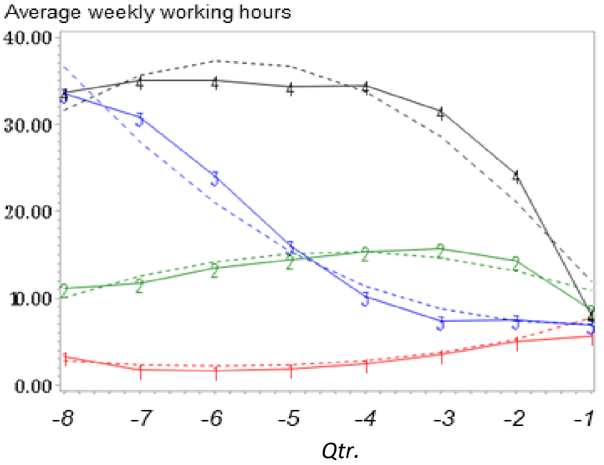

Five groups

Six groups

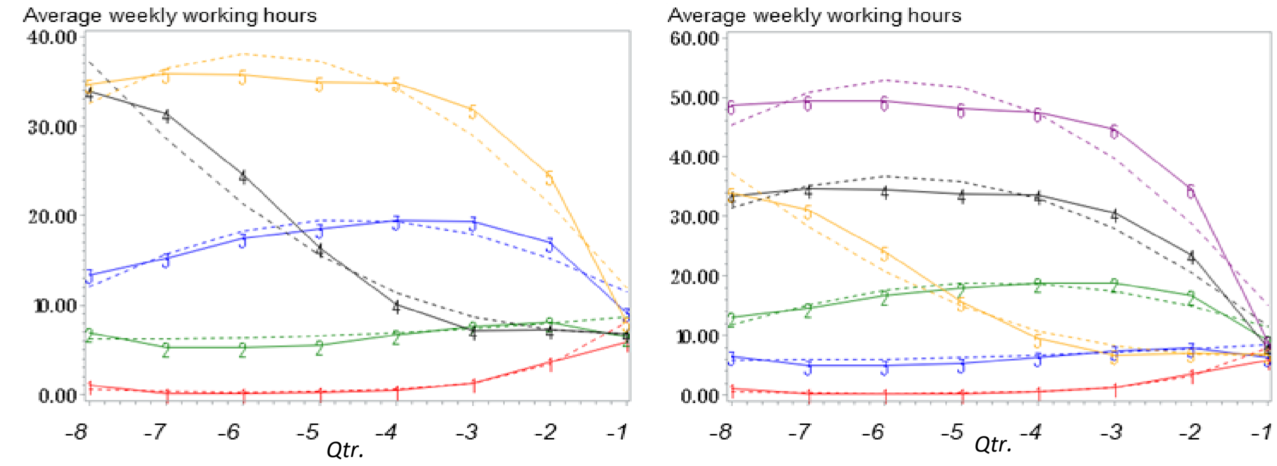

Seven groups

Eight groups

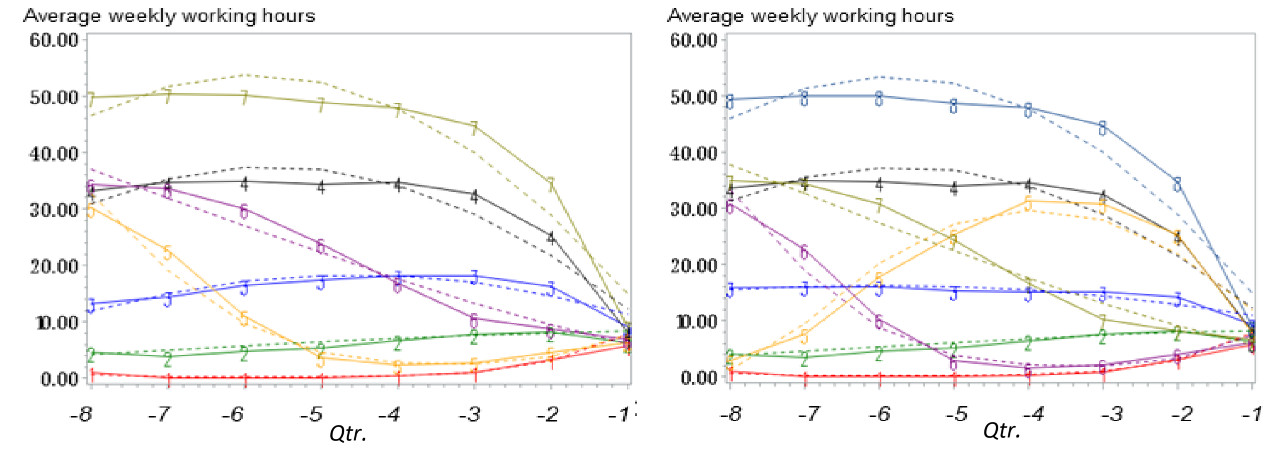

Nine groups

Ten groups

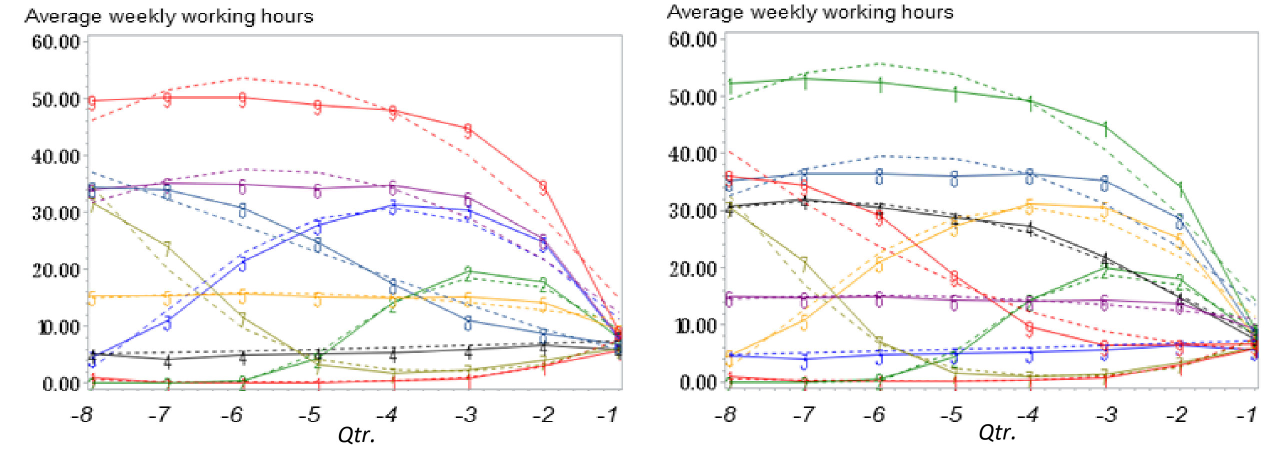

Figure A3.1-10: Different trajectory grouping

A3.2 Best-fit model

The BIC log Bayes factor approximation suggested using 10 trajectory groups. The average posterior probabilities of group membership were all above 0.8 in all models. Group sizes decreased with number of groups. The visual examination suggested using at least 8 groups, which allowed us to capture the fluctuating trajectory groups. We decided to use 10 trajectory groups for the retrospective marginal part-time work trajectories.

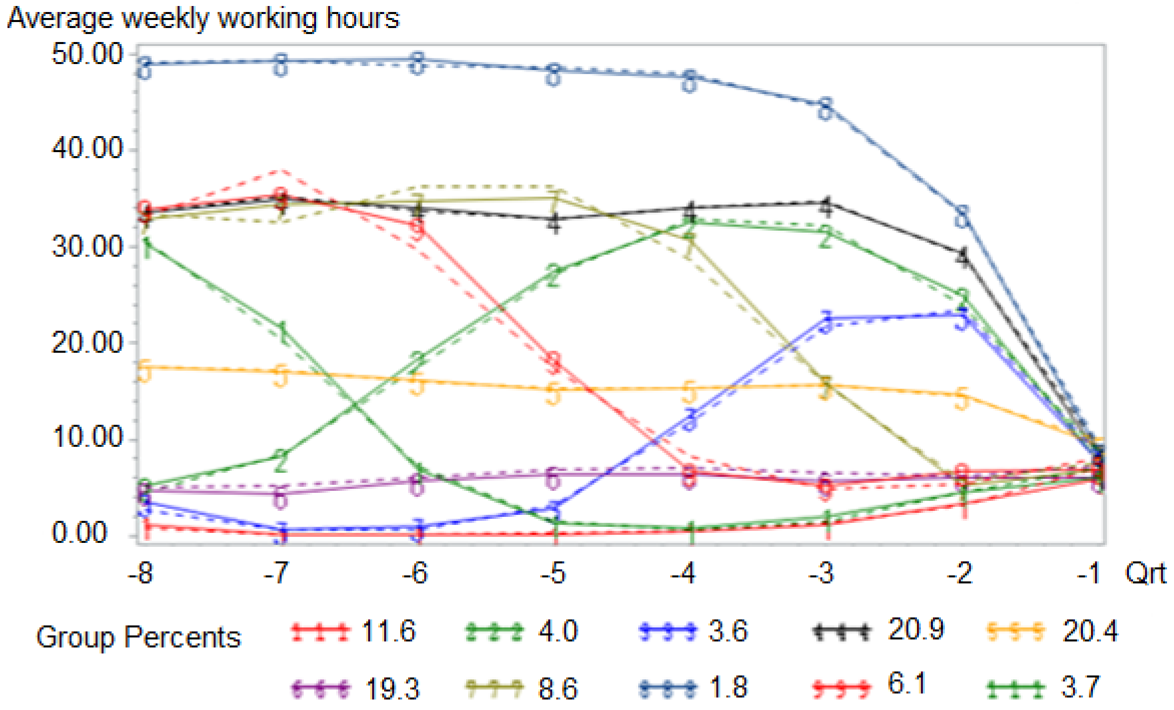

Figure A3.2 *Best-fit model for the retrospective marginal part-time work trajectories using 10 trajectory groups*

A3.3 Categorizing the retrospective trajectories

Table A3.3: Combining the 10 trajectories from the best-fit model for the retrospective marginal part-time work trajectories into three groups

Trajectory group nr.	Constant, N = 2011 (51.6%) 1/ 5/6		Mobile, N = 1594 (40.9%) 4/7/ 8/ 9/ 10		Fluctuating, N = 292 (7.5%) 2/ 3	
	Shape[A]	Avg grp prb [B]	Shape[A]	Avg grp prb [B]	Shape[A]	Avg grp prb [B]
1	1h-0h-6h	0.91/0.00/0.00		0.00/0.01/0.00/0.00/0.00		0.01/0.07
2		0.02/0.01/0.00		0.00/0.00/0.00/0.00/0.00	5h-27h-8h	0.87/0.10
3		0.01/0.00/0.07		0.00/0.01/0.00/0.00/0.00	3h-24h-7h	0.02/0.87
4		0.03/0.01/0.04	34h-33h-8h	0.79/0.00/0.12/0.04/0.00		0.00/0.00
5	18h-15h-9h	0.00/0.86/0.08		0.04/0.00/0.02/0.00/0.00		0.01/0.00
6	4h-6h-6h	0.00/0.02/0.87		0.02/0.01/0.00/0.02/0.00		0.00/0.07
7		0.00/0.00/0.03	33h-35h-7h	0.00/0.88/0.00/0.04/0.00		0.00/0.05
8		0.01/0.00/0.00	50h-49h-9h	0.09/0.00/0.90/0.00/0.01		0.00/0.00
9		0.00/0.00/0.05	34h-18h-7h	0.09/0.02/0.00/0.84/0.00		0.00/0.00
10		0.00/0.00/0.00	30h-1h-6h	0.00/0.00/0.13/0.00/0.87		0.00/0.00

[A] The median group values of working hours across three time points at qtr. -8, -4 and -1.

[B] The average posterior probabilities of group membership for each trajectory group. Within each category the probabilities of the belonging trajectory groups are presented separated with a "/".

Appendix 4: Supplementary analyses. The prospective marginal part-time trajectories

The prospective trajectories were identified among all employees start in marginal part-time at 2 years before baseline (at -qtr.8 in figure 2), N = 4.314 employees. Group based trajectories of prospective marginal part-time were calculated based on quarterly average weekly working hours across the two years leading up to baseline (in figure 2: from -8 qtr. to -1 qtr.).

A4.1: Flowchart of the prospective study population

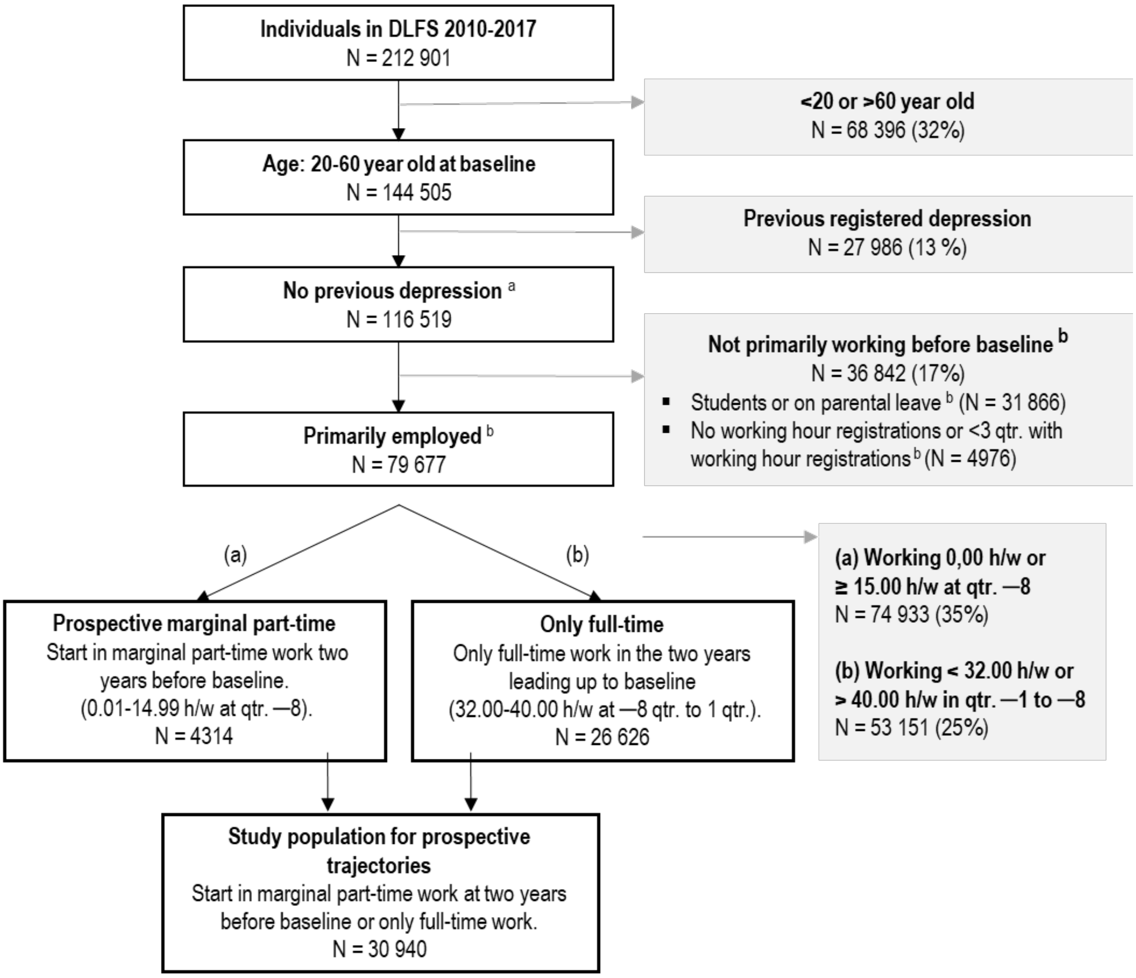

[a] No registration of a diagnosed depressive episode or purchasing of antidepressant drugs before baseline

[b] In the two years before baseline: Not receiving the State Educational Grant (S.U.), parental leave benefits or having no registered working hours

Figure A4.1: Flowchart of the prospective study population

A4.2 Different trajectory grouping of the prospective marginal part-time trajectories

---

One groups

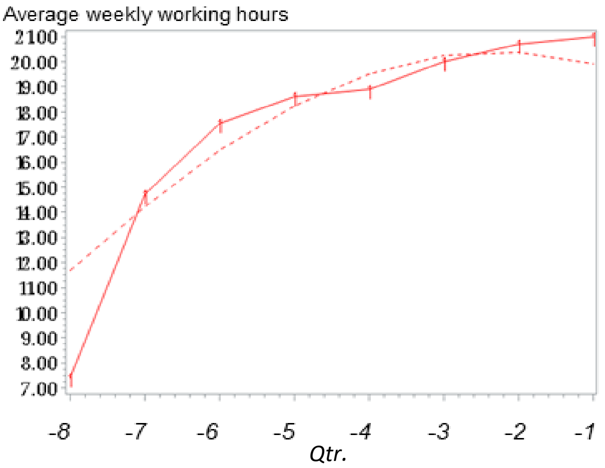

Two groups

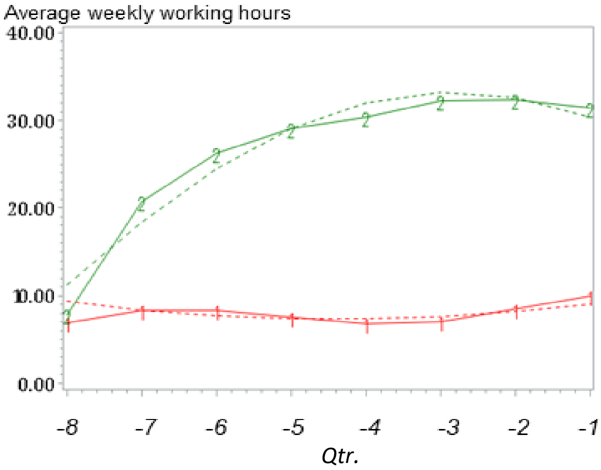

Three groups

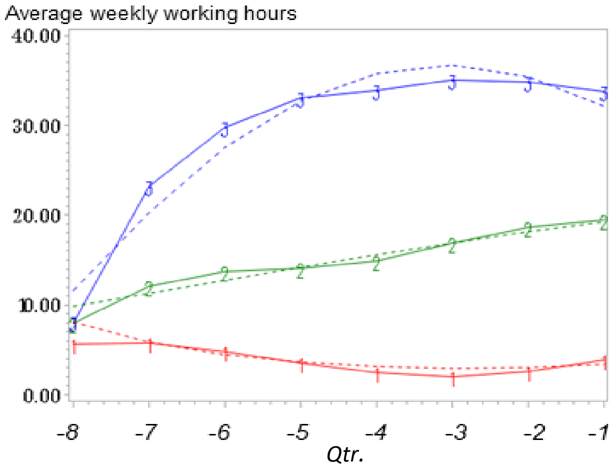

Four groups

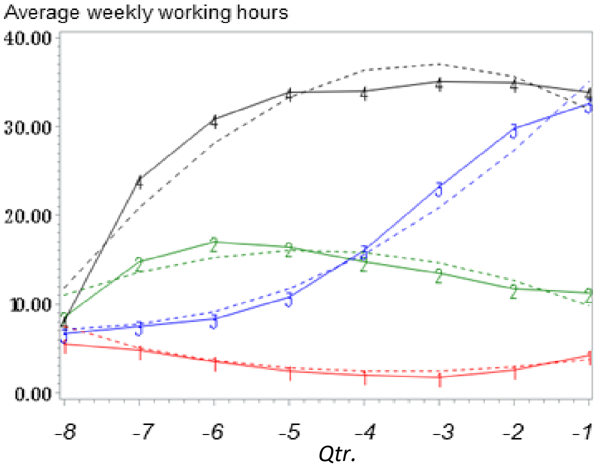

Five groups

Six groups

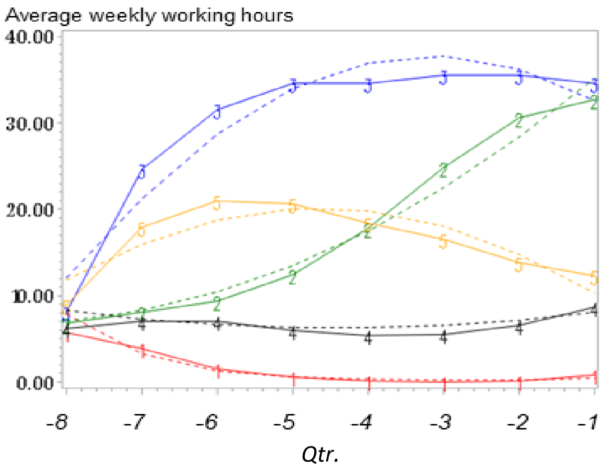

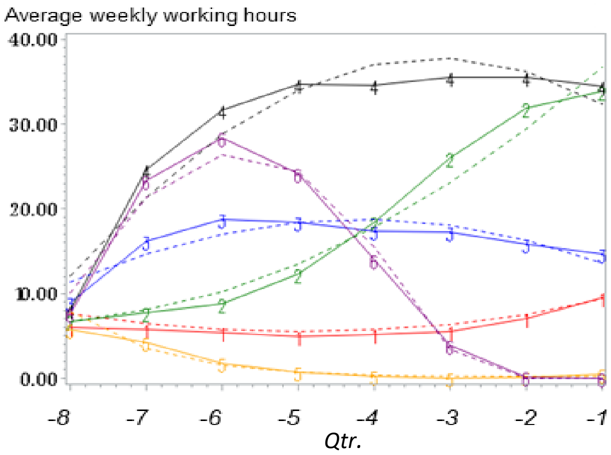

Seven groups

Eight groups

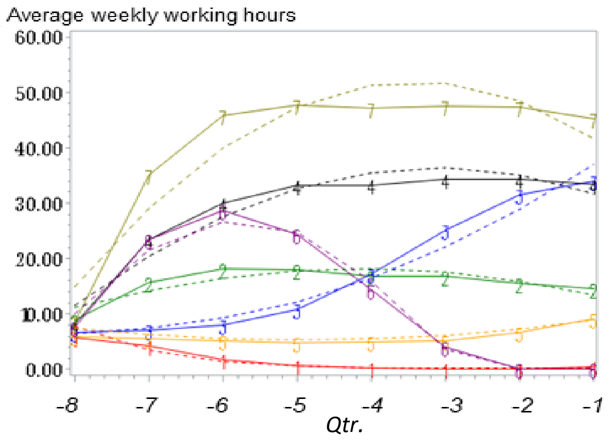

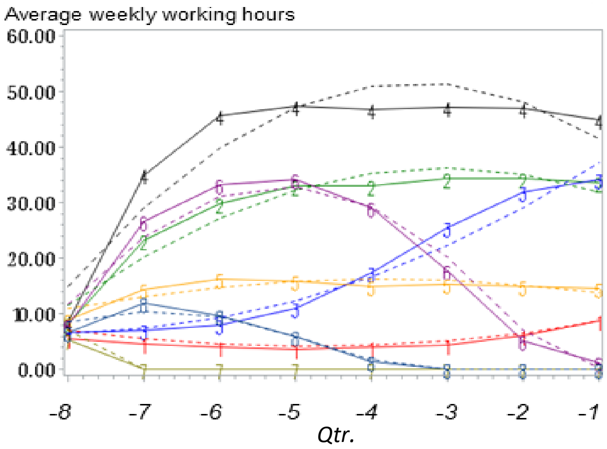

Nine groups

Ten groups

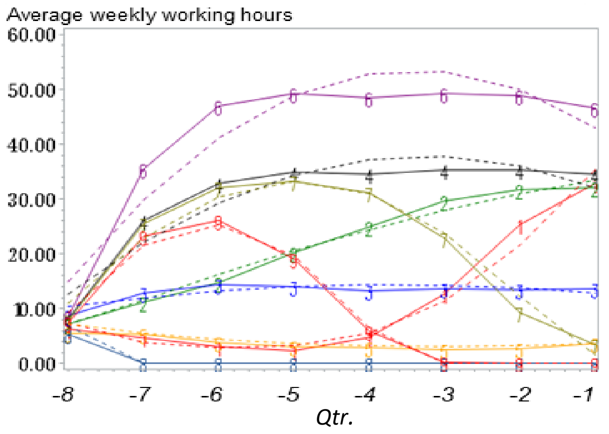

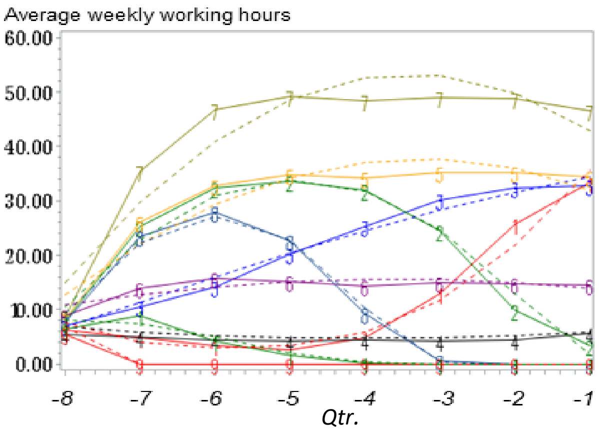

Figure A4.2.1-10: Different trajectory grouping

A4.3 Best-fit model

The BIC log Bayes factor approximation suggested using 10 trajectory groups. The average posterior probabilities of group membership were above 0.8 for all number of groups. The group size decreased with number of groups. The visual examination suggested using at least 6 groups, which allowed us to capture the Fluctuating trajectory groups. We decided to use 10 trajectory groups for the prospective marginal part-time work trajectories.

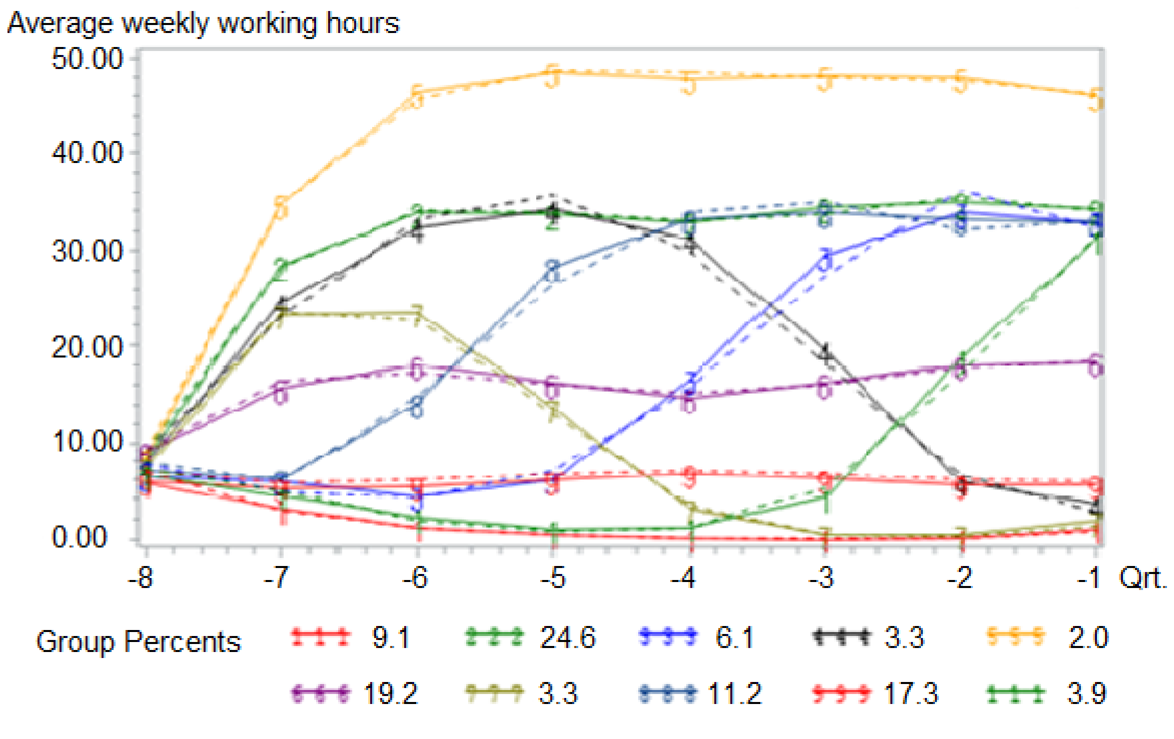

Figure A4.3 Best-fit model for the prospective marginal part-time work trajectories using 10 trajectory groups

A4.4: Categorizing the prospective trajectories

Table A4.4: Combining the 10 trajectories from the best fit model for the prospective marginal part-time work trajectories into three groups.

Trajectory group nr.	Constant, N = 2127(49.3%) 1/6/7/9		Mobile, N = 2051(47.5%) 2/3/5/8/10		Fluctuating, N = 136(3.2%) 4	
	Shape^A	Avg grp prb ^B	Shape^A	Avg grp prb ^B	Shape^A	Avg grp prb ^B
1	6h-0h-1h	0.94/0.00/0.01/0.05		0.00/0.00/0.00/0.00/0.00		0.00
2		0.00/0.01/0.00/0.00	8h-34h-34h	0.95/0.00/0.01/0.03/0.00		0.00
3		0.00/0.05/0.00/0.00	6h-6h-33h	0.00/0.88/0.00/0.06/0.02		0.00
4		0.00/0.03/0.01/0.00		0.01/0.00/0.00/0.00/0.00	8h-29h-2h	0.94
5		0.00/0.00/0.00/0.00	8h-49h-46h	0.08/0.00/0.92/0.00/0.00		0.00
6	9h-16h-18h	0.00/0.90/0.00/0.04		0.02/0.01/0.00/0.01/0.00		0.01

7	7h-14h-2h	0.01/0.01/0.90/0.06		0.00/0.00/0.00/0.00/0.00	0.01
8		0.00/0.02/0.00/0.00	7h-28h-33h	0.05/0.04/0.00/0.89/0.00	0.00
9	6h-6h-5h	0.02/0.05/0.01/0.91		0.00/0.00/0.00/0.00/0.00	0.00
10		0.01/0.01/0.00/0.04	7h-1h-31h	0.00/0.02/0.00/0.00/0.92	0.00

[A] The median group values of working hours across the time points (qtr: -8, -4, -1).

[B] The average posterior probabilities of group membership for each trajectory group. Within each category the probabilities of the belonging trajectory groups are presented separated with a "/".

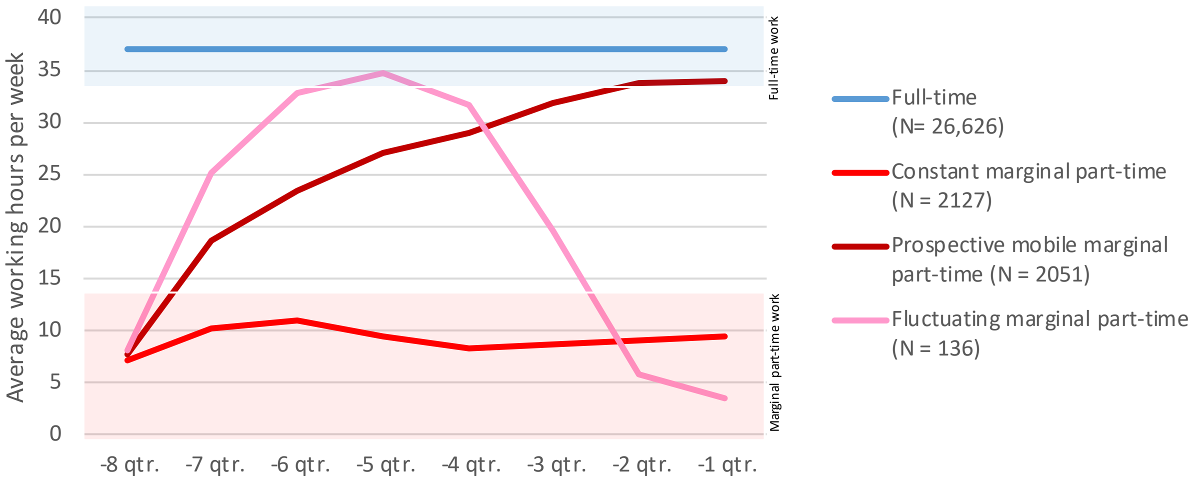

Figure A4.4: *The average number of weekly working hours in the two years leading up to baseline by each prospective trajectory group.*

A4.5 Results from the analyses using prospective trajectories of marginal part-time work.

During the two-year follow-up 1.5 % of the prospective study population experienced depression (475 cases), with 460 cases first identified from antidepressant redemption and 15 cases first identified from hospital diagnosis.

Table A4.5.1: Descriptive characteristic of the prospective study population at baseline by the three prospective trajectories of marginal part-time work and full-time work

	Prospective marginal part-time trajectories								Full-time workers	
	Constant		Prosp. mobile		Fluctuating		Total		Full-time	
	N	%	N	%	N	%	N	%	N	%
Sex										
Women	1118	52.6	942	45.9	62	45.6	2122	49.2	12 667	47.6
Age										
20-29	508	23.9	539	26.3	36	26.5	1083	25.1	1697	6.4
30-39	349	16.4	530	25.8	29	21.3	908	21.0	4524	17.0
40-49	481	22.6	483	23.6	31	22.8	995	23.1	9493	35.7
50-60	789	37.1	499	24.3	40	29.4	1328	30.8	10 912	41.0
Ethnicity										
Danish origin	1627	76.5	1661	81.0	108	79.4	3396	78.7	25 126	94.4
Immigrant or descendant	500	23.5	390	19.0	28	20.6	918	21.3	1500	5.6
Cohabitation										
Cohabitation	913	42.9	946	46.1	59	43.4	1918	44.5	17 599	66.1
No cohabitation	1214	57.1	1105	53.9	77	56.6	2396	55.5	9027	33.9
Morbidity										
Previous diagnosed illness[A]	217	10.2	128	6.2	12	8.8	357	8.3	1333	5.0
No diagnosed illness	1910	89.8	1923	93.8	124	91.2	3957	91.7	25 293	95.0
Education level										
Primary	596	28.0	406	19.8	33	24.3	1035	24.0	2712	10.2
Secondary	912	42.9	981	47.8	73	53.7	1966	45.6	13 182	49.5
Higher	499	23.5	608	29.6	26	19.1	1133	26.3	10 579	39.7
Industry										
Construction, industry, agriculture, professional service	584	27.5	751	36.6	55	40.4	1390	32.2	7264	27.3
Public administration, education, health and culture	829	39.0	744	36.3	46	33.8	1619	37.5	10 709	40.2
Finance, real estate, communication	108	5.1	113	5.5	<10	6.6	230	5.3	3198	12.0
Trade	455	21.4	439	21.4	25	18.4	919	21.3	5441	20.4

Years										
2010	316	14.9	319	15.6	31	22.8	666	15.4	3126	11.7
2011	259	12.2	285	13.9	18	13.2	562	13.0	3404	12.8
2012	282	13.3	266	13.0	19	14.0	567	13.1	3317	12.5
2013	256	12.0	249	12.1	14	10.3	519	12.0	3471	13.0
2014	282	13.3	249	12.1	15	11.0	546	13.7	3317	12.5
2015	302	14.2	292	14.2	12	8.8	606	14.0	3574	13.4
2016	430	20.2	391	19.1	27	19.9	848	19.7	6382	24.0
Family income										
1 quartile	1249	58.7	881	43.0	79	58.1	2209	51.2	4854	18.2
2 quartile	521	24.5	630	30.7	37	27.2	1188	27.5	6472	24.3
3 quartile	201	9.5	303	14.8	14	10.3	518	12.0	7521	28.3
4 quartile	156	7.3	237	11.6	<10	4.4	399	9.2	7779	29.2
Income insecurity										
yes	239	11.2	139	6.8	12	8.8	390	9.0	539	2.0
Job insecurity										
yes	395	18.6	242	11.8	14	10.3	651	15.1	194	0.7

Full-time work: continuously 32-40 hours of work per week across the past two years before baseline; Prospective trajectories: paths of working hours leading up to baseline, which starts in marginal part-time work (0.01-14.99 hours of work per week) at two years before baseline; Constant = stable trajectory with constant marginal part-time work. Prospective mobile = increasing trajectory going from marginal part-time work towards more hours of work. Fluctuating = oscillating trajectory fluctuating in and out of marginal part-time work. Prosp. = prospective. N = number of workers. % = percentage of workers. [A] within the past five year. Assessed by Charlson's comorbidity index

Table A4.5.2: The associations between the prospective trajectories of marginal part-time work and depression.

Trajectory	Person years	Cases of depression	Cases per 10 000 person years	Crude		Model 1		Model 2	
		N	N	HR	95% CI	HR	95% CI	HR	95% CI
Prospective trajectories (N = 30 940)									
Full-time work	52 613	341	64.8	1	.	1	.	1	.
Constant	4060	78	192.1	2.96	2.32–3.79	2.67	2.06–3.46	2.16	1.63–2.86
Prospective mobile	3976	46	115.7	1.78	1.31–2.43	1.59	1.15–2.20	1.44	1.03–1.99
Fluctuating	251	10	398.4	6.14	3.27–11.52	5.38	2.85–10.14	4.65	2.45–8.89

Trajectory: paths of working hours across the past two years before baseline; Full-time: 32-40 hours of work per week; Constant = Stable trajectory with constant marginal part-time work. Mobile = decreasing trajectory going from more hours of work towards marginal part-time work. Fluctuating = oscillating trajectory fluctuating in and out of marginal part-time work. N = number of workers. HR = Hazard Ratio. 95% CI = 95% confidence interval. Crude: no adjustment. Model 1: adjusted for age, sex, education and calendar year. Model 2: adjusted for model 1 and Industry, morbidity, cohabitation, ethnicity and Family income. N=number. HR = Hazard Ratios. 95% CI=95% confidence interval.

Appendix A4.5.3: Mediation analyses of respectively job insecurity or income insecurity in the relation between prospective marginal part-time work and depression (N = 30 940)								
Trajectory	Full-time		Marginal part-time					
			Crude		Model 1		Model 2	
	HR	95% CI	HR	95% CI	HR	95% CI	HR	95% CI
Job insecurity								
Natural direct	1	-	2.11	1.94-2.26	2.11	1.94–2.26	1.80	1.66–1.94
Natural indirect	1	-	1.06	0.96–1.19	1.06	0.96–1.19	1.05	0.93–1.16
Total	1	-	2.24	2.13-2.38	2.24	2.13–2.38	1.88	1.76–2.01
Mediated proportion[A]	-	-	7.6	-4.90–20.49	7.64	-4.89–20.49	6.76	-12.40–22.07
Income insecurity								
Natural direct	1	-	2.57	2.42–2.75	2.31	2.15–2.48	1.94	1.80–2.09
Natural indirect	1	-	0.97	0.88–1.06	0.97	0.88–1.07	0.97	0.87–1.07
Total	1	-	2.49	2.39–2.60	2.24	2.13–2.37	1.88	1.76–2.00
Mediated proportion[A]	-	-	-3.44	-14.20–5.88	-3.74	-15.79–8.58	-5.53	-24.5–10.54

Adjusted for model 2: age. sex. education. calendar year. industry. morbidity. cohabitation. heritage and Family income. Full = constant full-time work. MPT= combined group of the constant. mobile and Fluctuating trajectory groups of marginal part-time work. [A]The mediated proportion is calculated as the coefficients for the natural indirect (log(HR)) divided by coefficients for the total effect (log(HR)) .

Table A4.5.4: The association between prospective trajectories of marginal part-time and redeemed antidepressant drugs, during a two-year follow-up.									
Trajectory	Person years	Cases of depression	Cases per 10 000 person years	Crude		Model 1		Model 2	
		N	N	HR	95% CI	HR	95% CI	HR	95% CI
Redeemed antidepressant drugs									
Full-time work	52617	337	64.0	1	-	1	-	1	-
Constant	4061	76	187.1	2.92	2.28–3.75	2.62	2.02–3.41	2.15	1.61–2.86
Mobile	3977	45	113.2	1.77	1.29–2.41	1.58	1.14–2.18	1.43	1.03–1.99
Fluctuating†	-	-	-	-		-		-	

Trajectory: paths of working hours across the past two years before baseline. Full-time= 32-40 hours of work per week. Constant = Stable trajectory with constant marginal part-time work. Mobile = decreasing trajectory going from more hours of work towards marginal part-time work. Fluctuating = oscillating trajectory fluctuating in and out of marginal part-time work. N = number of workers. HR = Hazard Ratio. 95% CI = 95% confidence interval. Crude: no adjustment. Model 1: adjusted for age, sex, education and calendar year. Model 2: adjusted for model 1 and Industry, morbidity, cohabitation, ethnicity and Family income. Crude N = 30 940, Model 1 N = 30 607, Model 2 N = 30 448. †Results not shown due to too few cases

Results on hospital-diagnosed depression are not presented due to too few cases in the trajectory groups of marginal part-time work.

Table A4.5.5: The associations between prospective marginal part-time work and depression by sex and age, during a two-year follow-up. Sex: Women (N = 14,789) and men (N = 16,151). Age groups: 20-29 (N = 2,780), 30-39 (N = 2,662), 40-49 (N = 10,488), and 50-60 (N = 12,240).

		Trajectory	Person years	Cases of depression	Cases per 10 000 person years	Crude		Model 1		Model 2	
				N	N	HR	95% CI	HR	95% CI	HR	95% CI
Prospective trajectories											
Sex	Women	Full-time	25 034	203	81.1	1	.	1	.	1	.
		Marginal part-time	4078	73	179.0	2.20	1.69–2.88	2.00	1.50–2.66	1.73	1.28–2.34
	Men	Full-time	27 579	138	50.0	1	.	1	.	1	.
		Marginal part-time	4209	61	144.9	2.90	2.15–3.92	2.60	1.88–3.59	2.08	1.46–2.96
Age	20-29	Full-time	3293	21	63.8	1	.	1	.	1	.
		Marginal part-time	2041	35	171.5	2.69	1.57–4.63	2.80	1.62–4.83	2.56	1.45–4.51
	30-39	Full-time	8923	59	66.1	1	.	1	.	1	.
		Marginal part-time	1751	21	119.9	1.82	1.10–2.99	1.65	0.98– 2.79	1.52	0.86–2.67
	40-49	Full-time	18 785	121	64.4	1	.	1	.	1	.
		Marginal part-time	1914	40	209.0	3.24	2.27–4.63	2.67	1.83–3.90	1.87	1.24–2.83
	50-60	Full-time	21 611	140	64.8	1	.	1	.	1	.
		Marginal part-time	2582	38	147.2	2.27	1.59–3.25	2.13	1.47– 3.08	1.84	1.24–2.72

Trajectory: paths of working hours across the past two years before baseline; Full-time: 32-40 hours of work per week; Marginal part-time: All three prospective trajectories with marginal part-time (0.01-14.99 weekly working hours at two years before baseline), i.e. includes the constant, prospective mobile and fluctuating marginal part-time trajectory. N = number of workers. HR = Hazard Ratio. 95% CI = 95% confidence interval.
Crude: no adjustment. Model 1: adjusted for calendar year, education and age (in analyses stratified by sex) or sex (in analyses stratified by age). Model 2: adjusted for model 1 and industry, morbidity, cohabitation, ethnicity and Family income

Appendix 5: Post-hoc analyses

A5.1: The association between retrospective trajectories of marginal part-time and depression by redeemed antidepressant and hospital-diagnosed depression.

Results on hospital-diagnosed depression are not presented due to too few cases in the trajectory groups of marginal part-time work.

Table A5.1: The association between trajectories of marginal part-time and redeemed antidepressant drugs or hospital-diagnosed depression, during a two-year follow-up.

Trajectory	Person years	Cases of depression	Cases per 10 000 person years	Crude		Model 1		Model 2	
		N	N	HR	95% CI	HR	95% CI	HR	95% CI
Redeemed antidepressant drugs									
Full-time work	52617	337	64.0	1	-	1	-	1	-
Constant	3800	71	186.8	2.91	2.26–3.76	2.78	2.12–3.65	2.33	1.76–3.10
Mobile	3052	70	229.4	3.58	2.77–4.63	3.35	2.57–4.38	2.89	2.20–3.81
Fluctuating	552	16	289.9	4.52	2.74–7.46	4.31	2.56–7.27	3.59	2.12–6.10

Trajectory: paths of working hours across the past two years before baseline. Full-time = 32-40 hours of work per week. Constant = Stable trajectory with constant marginal part-time work. Mobile = decreasing trajectory going from more hours of work towards marginal part-time work. Fluctuating = oscillating trajectory fluctuating in and out of marginal part-time work. N = number of workers. HR = Hazard Ratio. 95% CI = 95% confidence interval. Crude: no adjustment. Model 1: adjusted for age, sex, education and calendar year. Model 2: adjusted for model 1 and Industry, morbidity, cohabitation, ethnicity and Family income. Crude N = 30 523, Model 1 N = 30 114, Model 2 N = 30 089.

A5.2: The association between retrospective marginal part-time and depression stratified by sex and age groups. The three trajectory groups: constant, mobile and fluctuating marginal part-time work are combined into one group (Marginal part-time).

Table A5.2: The associations between marginal part-time work and depression by sex and age, during a two-year follow-up. Sex: Women (N = 14,633) and men (N = 15,890). Age groups: 20-29 (N = 2,647), 30-39 (N = 5,229), 40-49 (N = 10,403), and 50-60 (N = 12,244).

		Trajectory	Person years	Cases of depression	Cases per 10 000 person years	Crude		Model 1		Model 2	
				N	N	HR	95% CI	HR	95% CI	HR	95% CI
Sex	Women	Full-time	25 034	203	81.1	1	-	1	-	1	-
		Marginal part-time	3 744	79	211.0	2.60	2.00–3.37	2.51	1.91–3.30	2.38	1.79–3.16
	Men	Full-time	27 579	138	50.0	1	-	1	-	1	-
		Marginal part-time	3 656	82	224.3	4.48	3.41–5.89	4.27	3.17–5.76	3.52	2.55–4.87
Age											
	20-29	Full-time	3 293	21	63.8	1	-	1	-	1	-
		Marginal part-time	1 761	24	136.3	2.13	1.19–3.83	2.11	1.15–3.86	2.20	1.18–4.08
	30-39	Full-time	8 923	59	66.1	1	-	1	-	1	-
		Marginal part-time	1 319	30	227.4	3.43	2.21–5.32	3.05	1.91–4.87	2.93	1.78–4.81
	40-49	Full-time	18 785	121	64.4	1	-	1	-	1	-
		Marginal part-time	1 735	55	317.0	4.92	3.57–6.76	4.14	2.94–5.82	3.22	2.24–4.62
	50-60	Full-time	21 611	140	64.8	1	-	1	-	1	-
		Marginal part-time	2 585	52	201.2	3.10	2.26–4.27	2.81	2.01–3.91	2.20	1.54–3.15

Trajectory: paths of working hours across the past two years before baseline; Full-time: 32-40 hours of work per week; Marginal part-time: All three retrospective trajectories with marginal part-time (0.01-14.99 weekly working hours at baseline), i.e. includes the constant, mobile and fluctuating marginal part-time trajectory. N = number of workers. HR = Hazard Ratio. 95% CI = 95% confidence interval.
Crude: no adjustment. Model 1: adjusted for calendar year, education and age (in analyses stratified by sex) or sex (in analyses stratified by age). Model 2: adjusted for model 1 and industry, morbidity, cohabitation, ethnicity and household income.

Appendix 6: Sensitivity analyses

All sensitivity analyses are based on the retrospective trajectory groups and adjusted for model 1.

A6.1 Non-response: We tested non-response in the DLFS, by using Statistic Denmark's population weights from the DLFS developed to ensure the DLFS sample is representative of the Danish population (11). See Table A6.1-2.

A6.2 No missing working hours: Group-based trajectory models assumed that data are missing at random, and handles missing data by fitting the model using maximum likelihood estimation. As such, we tested the results without missing values of working hours in the two years leading up to baseline. See Table A6.1-2.

A6.3 Combined mediator: To test if there was too little variance in the mediator insecurity variables, we analyses a combined mediator variable with either job insecurity and/or income insecurity. See Table A6.3-4.

A6.4 Constant trajectory: In addition, to test if the mediation is only present for the constant trajectory, which has the highest income and job insecurity, by using the combined insecurity mediator variable in the mediation analysis. See Table A6.3-4.

Table A6.1-2: Non-response or no missing working hours in the associations between trajectories of marginal part-time work and depression.

	Non-response (N = 30 114)		No missing working hours (N = 30 076)	
	HR	95% CI	HR	95% CI
Trajectory				
Full time	1	-	1	-
Constant	3.15	3.10–3.21	2.91	2.23–3.80
Mobile	3.47	3.41–3.53	3.32	2.54–4.33
Fluctuating	3.73	4.58–4.89	4.29	2.54–7.23

Retrospective trajectories. Adjusted for model 1: calendar year, education, age and sex.

Table A6.3-4: A combined mediator or only the constant marginal part-time work trajectory in the mediation analyses of marginal part-time work and depression.

	Natural direct		Natural indirect		Total		Mediated proportion	
	HR	95% CI	HR	95% CI	HR	95% CI	HR	95% CI
Combined mediator (N = 30 114)								
Full-time	1	-	1	-	1	-		
Marginal part-time	3.19	3.00–3.40	1.00	0.91–1.08	3.18	3.03–3.32	-0.41	-8.30–6.81
Constant trajectory (N = 28 282)								
Full-time	1	-	1	-	1	-		
Marginal part-time	2.90	2.71–3.10	0.96	0.85–1.08	2.80	2.55–3.05	-3.73	-17.48–7.29

Retrospective trajectories. Adjusted for model 1: calendar year, education, age and sex.

Appendix references

1. Textor J, van der Zander B, Gilthorpe MS, Liskiewicz M, Ellison GTH. Robust causal inference using directed acyclic graphs: the R package 'dagitty'. International Journal of Epidemiology. 2016;45(6):1887-94.
2. Salk RH, Hyde JS, Abramson LY. Gender differences in depression in representative national samples: Meta-analyses of diagnoses and symptoms. Psychol Bull. 2017;143(8):783-822.
3. Lorant V, Deliege D, Eaton W, Robert A, Philippot P, Ansseau M. Socioeconomic inequalities in depression: a meta-analysis. Am J Epidemiol. 2003;157(2):98-112.
4. Wulsin L, Alterman T, Timothy Bushnell P, Li J, Shen R. Prevalence rates for depression by industry: a claims database analysis. Soc Psychiatry Psychiatr Epidemiol. 2014;49(11):1805-21.
5. Vilhelmsson A. Depression and antidepressants: a nordic perspective. Front Public Health. 2013;1:30.
6. Moussavi S, Chatterji S, Verdes E, Tandon A, Patel V, Ustun B. Depression, chronic diseases, and decrements in health: results from the World Health Surveys. Lancet. 2007;370(9590):851-8.
7. Giannelis A, Palmos A, Hagenaars SP, Breen G, Lewis CM, Mutz J. Examining the association between family status and depression in the UK Biobank. Journal of Affective Disorders. 2021;279:585-98.
8. Charlson M, Szatrowski TP, Peterson J, Gold J. Validation of a combined comorbidity index. J Clin Epidemiol. 1994;47(11):1245-51.
9. Charlson ME, Pompei P, Ales KL, MacKenzie CR. A new method of classifying prognostic comorbidity in longitudinal studies: development and validation. Journal of chronic diseases. 1987;40(5):373-83.
10. Lynge E, Sandegaard JL, Rebolj M. The Danish National Patient Register. Scand J Public Health. 2011;39(7 Suppl):30-3.
11. Statistics Denmark. Labour Force Survey (LFS) [internet]. Copenhagen: Statistics Denmark; [cited 2022 Dec 20]. Available from: https://www.dst.dk/en/Statistik/dokumentation/metode/aku-arbejdskraftundersoegelsen.
